# Medical genetics and genomic medicine in Greece: achievements and challenges

**DOI:** 10.1002/mgg3.179

**Published:** 2015-09-15

**Authors:** Irini Manoli, Helen Fryssira

**Affiliations:** 1 Genetics and Molecular Biology Branch, National Human Genome Research Institute, National Institutes of HealthBethesda, Maryland; 2 Medical Genetics, Choremio Research Laboratory, “Aghia Sophia” Children’s Hospital, University of Athens – School of MedicineAthens, Greece


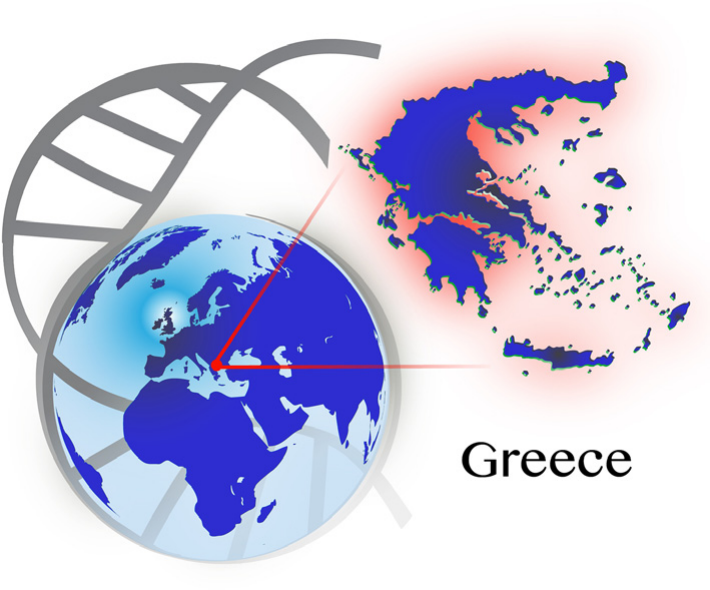


## Demographic Features

**Figure 1:**
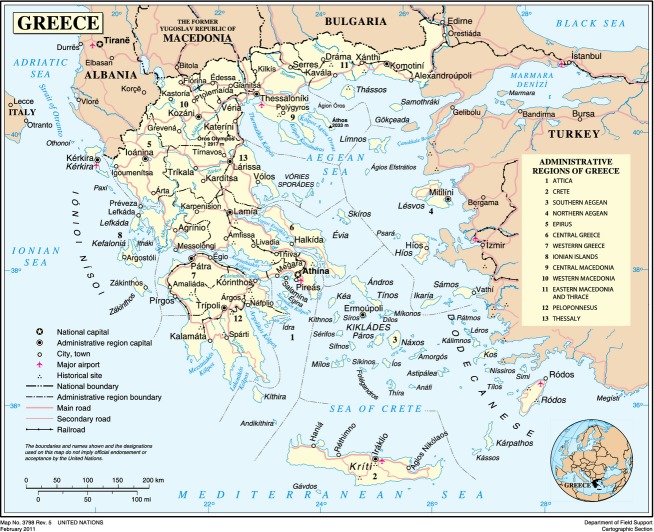
Map of Greece (Source: United Nations).

Greece (also known as Hellas) is located in southeastern Europe and is bordered on the north by Albania, the Former Yugoslav Republic of Macedonia (F.Y.R.O.M.) and Bulgaria; on the east by the Aegean Sea and Turkey; and on the west and south by the Ionian and Mediterranean seas, respectively. The country consists of the mainland, the Peloponnese peninsula and more than 3000 islands, of which about 170 are inhabited, including Crete, Rhodes, and Corfu, and the Ionian, Dodecanese, and Cyclades groups (Fig.1).

According to the most recent official consensus in 2011, the population was 10,816,286 (www.statistics.gr), with the vast majority being of Greek descent (98%) and the remainder belonging to other ethnicities. Over 95% of Greeks are Orthodox Christians. Muslims comprise 1.3% of the population and the remaining 0.7% includes Catholics, Jews, Old Calendar Orthodox, Jehovah Witnesses, Protestants, and other religions. The majority of the Muslim minority is concentrated in the area of northern Greece called Thrace. They are the only officially recognized minority in Greece and received legal status through the Treaty of Lausanne in 1923. Nearly half of the population lives in the two major cities, Athens and Thessaloniki, after a significant urbanization trend that was observed in the 20th and 21st centuries.

In 1981, Greece joined the European Union, and then it became the 12th member of the European Economic and Monetary Union in 2001. Greece being a gateway to Europe from the Middle East and Southwest Asia has experienced a rapidly growing number of immigrants/refugees due to the recent instability and conflicts in these regions, but has minorities also from eastern and central Europe, primarily Albania, Bulgaria, and other countries (2011 consensus).

## Genetics in Ancient Greece

Studies, theories, and observations on the inheritance of physical traits in humans can be traced back in ancient Greek literature from the eighth to the fourth century BC (Bazopoulou-Kyrkanidou 1992; McKusick 2007; Motulsky 2010). The data suggest that the development of genetic ideas started with the praise of the heroes’ noble origin in Homer’s epic poems (Bazopoulou-Kyrkanidou 1992). The concern about the lineage of the tragic figures in Greek drama and the theories on heredity and procreation expressed by the ancient physicians and philosophers helped influence the development of genetics for many centuries. Scientific evidence for patterns of genetic inheritance did not appear until Mendel’s work. However, history shows that humankind must have been interested in heredity long before the dawn of civilization, when the first speculations on heredity were recorded by the ancient Greeks (McKusick 2007; Motulsky 2010). Early Greek philosophers and physicians made observations on the inheritance of physical traits in humans, developed theoretical concepts, and proposed eugenic methods (Galton 1998).

Hippocrates (460–377 BC), the father of medicine, believed in the inheritance of acquired characteristics so he devised the hypothesis known as “pangenesis.” He proposed that all organs of a parent’s body gave off invisible seeds, which were like small building components transmitted during procreation to help the formation of the unborn child.

“Euploidy,” “euchromatin,” and “eugenics” begin with the Greek preface “eu” which means good/well. Every word in the ancient and modern Greek language which begins with “eu” implies something good (Galton 1998). Plato (427–347 BC) suggested “eugenic” policies, in order to supply the city–state with the finest possible progeny, with bodily and ethically eminent personalities, which was of vital significance for the city of Athens since they were constantly at war with other city–states. Plato’s methods to create an elite class, although discriminatory, are in accord with modern genetic theories.

Aristotle (384–322 BC), who was a student of Plato, developed a theory of inheritance, according to which each sex contributes to heredity, so that daughters resemble their mothers and sons their fathers. This concept was expounded through many generations. He also emphasized the importance of blood in heredity. One of his theories was that blood supplied genetic material for building all parts of the adult body and was the basis for passing on the power to the next generation. Today, people still speak of certain traits as being “in the blood” or “of blood lines” or “blood ties.” Furthermore, Aristotle was a central figure in our knowledge of ancient theories of reproduction/conception in animals, which were also applicable to humans. Democritus (460–370 BC) introduced the notion of nature–nurture when he wrote: “more people become capable by exercise than by their natural predisposition.”

Cyclopia is one of the severe forebrain lesions. Cyclops appears in Homer’s Odyssey (eighth to seventh century BC). The next time Cyclops appears in literature is in “Theogonia” written by Hesiodus (seventh century BC) and a satyric play of Euripides which is titled “Cyclops” (fifth century BC) (Kalantzis et al. 2013). A great number of descriptive terminology used in dysmorphology originate from the Greek language, including dysmorphology itself. Words like blepharophimosis, microphthalmia, aniridia, proboscis, synophrys, microcephaly, craniosynostosis, philtrum, ichthyosis, eczema, erythroderma, hyperkeratosis, epidermolysis, scleroderma, hypertrophy, anosmia, ataxia, myotonia, myopathy, osteopetrosis, and exostoses are only a few examples. Also, modern genetic terms such as “cloning” (from the Greek word “clonos” meaning “twig”) (Diamandopoulos and Goudas 2000), chromosome, gene, karyotype, trisomy, aneuploidy, homozygote, and others have Greek origin.

## Health Services in Greece

Greece has a National Health Service (NHS) founded in 1983, which is financed by the state budget, social insurance contributions, and private payments (Economou 2010). Various social insurance funds coexist with the National Health System (NHS). Some patients have private insurance to cover services in Greece or overseas. There are also private and public maternity hospitals. The cost of prenatal diagnosis and genetic testing, if needed, are in part covered by the public health insurance. In the Greek Constitution, health is considered a social right. There is free access through NHS to health centers and hospitals for the poor.

Since July 2011, increasing austerity measures resulted in citizens being forced to contribute more toward the cost of their healthcare services and medication. Insufficient public funding has forced households to look for care in the private sector (Siskou et al. 2009). Immigrants who are documented and legal residents in Greece are entitled to the same access to health care as Greek citizens. Formal access to the free services of the National Health System is dependent on registered employment and legal status. Undocumented migrants are only entitled to access hospital emergency services for the treatment of life-threatening conditions until their health has stabilized.

## Genetic Services in Greece

Cytogenetics departments in two of the main University hospitals in Athens, the “Aghia Sophia” Childrens’ Hospital and the “Alexandra” Maternity Hospital were the first to offer genetic services in the early 1960s. The Institute of Child Health was founded in 1965 as a diagnostic laboratory for metabolic disorders. It subsequently became the main laboratory in the country dedicated to newborn screening, which officially commenced in 1973 initially for PKU phenylketonuria and subsequently for G6PD glucose-6-phosphate dehydrogenase deficiency, galactosemia, and hypothyroidism. The program was based on the voluntary participation of national and private maternity units. Metabolic disorders clinical services are provided primarily at the First Department of Pediatrics of the University of Athens at the “Aghia Sophia” Children’s Hospital and only few other large academic centers.

Prenatal diagnosis was pioneered by the First Department of Pediatrics of the University of Athens at the “Aghia Sophia” Children’s Hospital, Choremion Research Laboratory in 1976, which was the first to diagnose chromosomal disorders. Chorionic villus sampling for prenatal diagnosis became available in 1983.

The Department of Medical Genetics (DMG) at the Choremion Research Laboratory of the First Department of Pediatrics of the University of Athens has the leading role in introducing modern molecular genetics methods for the diagnosis, carrier screening, and prenatal diagnosis of most genetic disorders in Greece. As the only major public, academic medical genetics center for the whole of the Hellenic area, DMG is also active in teaching and research. Located within the largest pediatric hospital in Greece (“Aghia Sophia” Children’s Hospital) it receives a large number of referrals from the hospital clinics, as well as outside referrals from Greece and other Balkan countries. Annually these include (approximately):

- 5000 samples for laboratory analysis
- 5000 cases for clinical evaluation and/or counseling

The genetic services include:

1. Clinical genetics and dysmorphology as an outpatient clinic offers:
  - Patient evaluation (diagnosis, prognosis, prevention, and risk assessment)
  - Genetic counseling
2. Our well-equipped Genetics Laboratory provides:
  - Molecular genetics
  - Cytogenetics (pre- and postnatal)
  - Molecular cytogenetics (pre- and postnatal)
  - Prenatal diagnosis (monogenic diseases and karyotype)
  - Preimplantation genetic diagnosis
  - Noninvasive prenatal diagnosis (sex and rhesus)
  - Microarrays technologies for CGH (Comperative Genomic Hybridization) and expression studies

Concurrent to the major role that the DMG plays in clinical services, it has also made substantial contributions in several areas of applied research in the field of genetics. Public funding for genetic testing outside a standard karyotype is limited and the patient’s family usually pays the remainder of the genetic, metabolic, and enzymatic testing privately.

**Figure 2:**
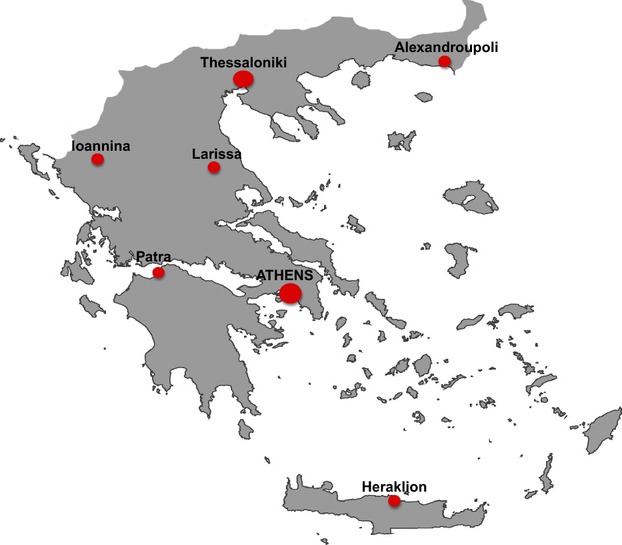
Genetic academic departments.

A number of other public genetic centers appeared in the 1990s at the Universities of Thessaloniki, Ioannina, Patras, Larissa, Alexandroupoli, and Crete (Fig.2). Currently these centers collectively offer prenatal diagnostic services, in addition to thalassemia, cystic fibrosis, muscular dystrophies, and for other neuromuscular disorders, fragile X, neurological and mitochondrial disorders, Wilson disease, polycystic kidney disease, Prader–Willi/Angelman syndromes, Williams syndrome, Rett syndrome, congenital heart disease, and many others (Kanavakis et al. 2003; Amenta et al. 2004; Yiallouros et al. 2007; Sofocleous et al. 2008, 2009; Zappu et al. 2008; Sakellariou et al. 2012; Syrmou et al. 2013; Kekou et al. 2015).

There are official referral centers for hemoglobinopathies across the country offering patient treatment, carrier screening, and prenatal diagnosis.

There is no official recognition of laboratories to perform diagnostic genetic tests, quality assessments, or accreditation for genetics services. In recent years though, several molecular genetics laboratories have joined the quality assessment programs of EMQN and one cytogenetics laboratory has joined the UK NEQAS in clinical cytogenetics. Also, the DMG at the Choremion Research Laboratory is a member of the UK NEQAS for Molecular Genetics, Special Advisory Group for external quality assessment (EQA) for Preimplantation Genetic Diagnosis. There are over 24 public and private institutions currently offering a variety of genetic services in Greece, with ∼17 entries for molecular DNA diagnostic laboratories in the European Directory of DNA Diagnostic Laboratories (EDDNAL) website.

The number of trained physicians in Clinical and Biochemical Genetics is extremely limited. Contacts and scientific collaboration with various foreign countries have provided an avenue for further exploration of genetic disorders. Clinical Genetics has not as yet been recognized as a separate specialty by the Hellenic Ministry of Health to date. Clinical dysmorphology services exist as a consulting and outpatient service at the “Aghia Sophia” Children’s Hospital in Athens, Choremion Research Laboratory, but are almost nonexistent elsewhere in Greece (Fryssira et al. 1997, 2015). Public funding for genetic testing outside a standard karyotype is limited and the patient’s family usually pays the remainder of the genetic, metabolic, and enzymatic testing privately.

Surveys of representative samples of the population in 1998 (Mavrou et al. 1998) and 2010 (Kitsiou-Tzeli et al. 2010) showed lack of awareness and acceptance of prenatal diagnostic procedures in low social–economical and educational backgrounds or in recent years in immigrant families (Karagkiouzis et al. 2015).

## Diagnosis, Treatment, and Prevention of Hemoglobinopathies

Greece has played a pioneer role in the field of hemoglobinopathies, both in the diagnosis, clinical management, therapeutic trials, prenatal diagnosis, and research, while the National Thalassemia Prevention Program has effectively decreased the incidence of thalassemia major and sickle cell (SC) syndromes in Greece.

Thalassemias are the most frequent genetic disorders in Greece. *β*-Thalassemia (*β*-thal) carrier frequency is ∼8%, while 1.5% of the population are carriers of the HbS [*β*6(A3)Glu→Val] mutation, sickle cell disease (SCD). The rate of *β*-thal carriers can be as high as 15–20% in some areas, especially in the low-altitude areas of Thessaly, Western Peloponnese, and Western Epirus. The distribution of HbS shows a similar pattern with certain regions, such as Viotia, western Peloponnese, and the Chalkidiki peninsula reaching a carrier frequency of up to 20%.

This resulted in the interest of the international scientific community regarding the clinical symptoms, the pathophysiological findings, and the inheritance of these diseases. For many decades, these served as models for the study of monogenic diseases on a clinical, biochemical, and later at a molecular level.

The Greek program for thalassemia started in 1974 as a nationwide government-sponsored and prospectively organized program, the National Program for the Prevention of Haemoglobinopathies, and is implemented free of charge through a network of 23 Prevention Units distributed across the country. All procedures are coordinated and supervised by the Central Prevention Unit at Laikon Hospital in Athens.

The first cases of prenatal diagnosis for hemoglobinopathies in Greece were carried out in 1975. In the early cases, the presence of two thalassemia genes in the fetus was determined by globin biosynthesis of a minute amount of fetal blood obtained through a fetoscope at the 18–19th week of pregnancy; the aim was to measure the newly formed *β*-globin chains, which had to exceed an empirically set level. This was a delicate step as in Greece most fetuses with thalassemia major are *β*^+^/*β*^+^ and therefore displayed values that could overlap with those of carriers. The procedure has been performed since then in thousands of cases (Jensen et al. 1979; Aleporou-Marinou et al. 1980; Loukopoulos et al. 1982). Today, prenatal diagnosis is carried out on fetal DNA extracted from chorionic villi cells obtained at the 8–10th week of pregnancy.

Based on the annual rates over the past 35 years, ∼12,000 couples at risk have had a healthy child. The program has managed to significantly reduce the number of new cases of thalassemia major and direct the available resources toward the optimization of treatment of the surviving patients (Voskaridou et al. 2012). The reduction in new cases was 81.1% and 84.6% for *β*-thal and SC, with a constant declining trend recorded over a 30-year period for *β*-thal, whereas for SCD, a transient reversal was observed in the mid-1990s probably due to an influx of immigrants of African origin (Ladis et al. 2013).

In recent years, hemoglobinopathies have become one of the most common applications for preimplantation diagnosis for monogenic disorders, as documented in the European Society of Human Reproduction and Embryology (ESHRE) annual reports (Harper et al. 2012; Moutou et al. 2014; Traeger-Synodinos et al. 2011). Noninvasive prenatal diagnosis remains challenging but has been employed with success in some studies and certainly holds great promise for the future (Chiu et al. 2002; Li et al. 2005; Kolialexi et al. 2007; Papasavva et al. 2013; Traeger-Synodinos 2013).

## The Roma Population

Greek Roma citizens represent a vulnerable group with largely unexplored genetic diseases. The “Athigani” as they are called in Greece are descendants of the Indian tribe “Romni” that left their country in the 10–12th— century (Mendizabal et al. 2011). A subgroup travelled through the Bosporous straits into Greece and the Balkan Peninsula. They landed in Crete in 1322 and Corfu in 1346 (Bartsocas et al. 1979). It is important to separate them from another group, named “Gypsies,” that appeared in Greece in the ninth century through Egypt.

Significant difficulties in access to healthcare services by both these minorities, result from the fact that the majority of these populations do not have identity or citizenship documents and they travel frequently. They often have consanguineous marriages and live in isolated settlements.

Because of the high incidence of inbreeding in these populations a high rate of genetic and metabolic disorders is to be expected. Unique phenotypes have been described, such as congenital cataracts, facial dysmorphism, neuropathy syndrome, an autosomal recessive syndrome caused by a common homozygous mutation in the *CTDP1* gene, which are exclusively manifested in the Roma population (Tzifi et al. 2011). A common founder homozygous C283Y mutation in *LGMD2C* γ-sarcoglycan gene has been described in gypsy families from different European countries, including Greece (Spengos et al. 2010).

Some limited preventive services are offered to the Roma population by regional health authorities in cooperation with the Ministry of Health, such as a comprehensive immunization program, which is offered via mobile health units that visit Roma camps and provide health promotion and preventive services.

## Other Isolates

There are several other isolated cohorts in mountainous areas of northern Greece (Pomaks) or Crete that represent genetic isolates and have allowed studies on genetic drift of certain disease-associated variants. Mutation analysis was performed in 110 and nine Wilson disease families originating from two isolated populations in Sardinia and the Greek Island of Kalymnos (Zappu et al. 2008), respectively. Ongoing research is also being carried out on some Greek islands for FSHD (Facioscapulohumeral muscular dystrophy).

## Academic Programs in the DMG

Some of the programs are:

1. Clinical trials run under the auspices of ESHRE entitled “The ESHRE Study into the evaluation of oocyte euploidy by microarray analysis (ESTEEM) trial,” 2012–present.
2. The TREAT-NMD rare disease registries for DMD (Duchenne muscular dystrophy) (Bladen et al. 2015)
3. The first Orthopedic Clinic and the DMG at the Choremion Research Laboratory of the University of Athens will participate in the European Reference Network for the following diseases: skeletal dysplasias, osteochondrodysplasias, osteogenesis imperfecta, achondroplasia.

## Patient Support Groups and Medical Associations for Genetics

The DMG at the Choremion Research Laboratory of the University of Athens organized in collaboration with the MDA Hellas (Muscular Dystrophy Association), a Greek-wide registry of patients diagnosed with a neuromuscular disease (www.hndr.gr).

The Associations for Disabled People include voluntary organizations and nongovernmental organizations (NGOs): Praksis, Doctors of the World, UNICEF, Médecins Sans Frontières, the Hellenic Society for the Protection and Rehabilitation of Disabled Children (ELEPAP), and the Red Cross are the most well known.

There are centers for Social Support for People with Disabilities such as the K.A.A.K.Y.A.MEA and the National Institution for the Rehabilitation of Disabled People (E.K.A.).

Various support groups for all different genetic diseases exist. “To Mellon” (“The Future” – www.tomellon.com.gr) is one of these, which provides social and financial support to parents with children suffering from genetic syndrome with or without intellectual disabilities. It also organizes seminars and meetings in order to deliver scientific information to professionals, members, and the general public.

Nonprofit volunteer groups offer support for specific genetic diseases such as cystic fibrosis, neurofibromatosis, Prader–Willi, the Panhellenic Union for Rare Diseases (P.E.S.P.A) which is the representative of Eurordis in Greece and many others. All these groups cooperate with professional medical staff.

In 1982, the Hellenic Association of Medical Geneticists (Σ.Ι.Γ.Ε.) was founded and consisted of professionals working in the field of medical genetics. This was a historical event in the development of Genetics in Greece. In January 2011, the Hellenic Society of Medical Genetics (HSMG) was established. Its aim was to unite all the medical doctors involved with genetic diseases and help the recognition of the specialty of Clinical Genetics in Greece.

## Challenges Ahead

In the last 10 years, Greece has gone forward in leaps and bounds in the fields of clinical genetics/dysmorphology, laboratory testing, and research. It has now introduced “state-of-the-art” technologies in diagnostic and prenatal testing and has success in the prevention of hemoglobinopathies. Some areas have difficulties in accessing highly specialized centers of clinical genetic services. Participation in extended newborn screening, expensive molecular genetic testing, especially whole exome sequencing, and costly therapies, such as enzyme replacement or gene and stem cell therapies, are difficult to cover by health insurances. The financial crisis has posed a burden in the services and support programs provided by the National Health System, with charities and the private sector trying to fill the void.

Academic institutions will have to play a critical role in public education in genetic disorders that will help improve their quality of life and social integration. Multidisciplinary care for the long-term needs of patients with complex genetic syndromes is difficult in urban areas, due to small numbers of genetically trained physicians. Access to information and genetic care is particularly challenging in rural areas, such as mountainous regions and smaller islands. Telemedicine has gained support and limited funding and could provide an avenue of sharing expertise in remote areas of the country.

There are patients with genetic diseases in all fields of Medicine. The contribution of Clinical Genetics is significant and essential for the diagnosis, prognosis, prevention, and risk assessment of genetic disorders. The correct evaluation of the clinical symptoms and of the morphological abnormalities constitutes the basis for accurate laboratory genetic testing and treatment in daily clinical practice. In Greece, the availability of genetic testing has expanded dramatically. Recently, whole exome or whole genome sequencing has begun to help solve diagnostic problems.

A major aim of the Greek genetics community is to have Clinical Genetics recognized as an independent Medical Specialty in Greece. In this way, we hope to (1) encourage young doctors to be trained and gain experience for the benefit of the patients; (2) promote scientific research in the field of medical genetics; (3) develop and specify what is necessary for the foundation of certified medical genetic centers; (4) organize quality control of the genetic services; and finally (5) gain recognition of medical geneticists as scientific professionals.
